# Long-term antihypertensive effect of a soluble cocoa fiber product in spontaneously hypertensive rats

**DOI:** 10.3402/fnr.v60.29418

**Published:** 2016-05-20

**Authors:** Sandra Fernández-Vallinas, Marta Miguel, Amaya Aleixandre

**Affiliations:** 1 Dpto. Farmacología, Fac. Medicina, U. Complutense, Madrid, Spain; 2 Instituto de Investigación en Ciencias de la Alimentación (CSIC-UAM, CEI+UAM), Madrid, Spain

**Keywords:** angiotensin-converting enzyme, antioxidant properties, blood pressure, fiber, spontaneously hypertensive rats

## Abstract

**Background and Methods:**

This study evaluates the antihypertensive effect of long-term intake of a soluble cocoa fiber product (SCFP). Different doses of SCFP were evaluated (200, 400, and 800 mg/kg/day) and a dose of 800 mg/kg/day of beta-glucan 0.75 (BETA-G) was used as a standard fiber. Water, a neutral vehicle, was used as negative control, and 50 mg/kg/day captopril was used as positive control. Systolic blood pressure (SBP) was measured weekly by the tail cuff method. Body weight, food, and liquid intake were also registered weekly in the rats from 10 to 24 weeks of life. Glucose, total cholesterol, and triglyceride levels; redox status; and the angiotensin-converting enzyme activity were also studied in the plasma samples of these animals.

**Results:**

Throughout the 10 weeks of treatment, captopril and SCFP (400 mg/kg/day) demonstrated blood pressure lowering effects in the spontaneously hypertensive rats (*p*<0.05; *n*=8). Paradoxically, neither the highest dose (800 mg/kg/day) of SCFP decreased SBP nor 800 mg/kg/day BETA-G (*p*>0.05; *n*=8). When the corresponding antihypertensive treatment, was disrupted the SBP values of the 400 mg/kg/day SCFP treated animals returned to control values (*p*>0.05; *n*=8). In addition, the SCFP significantly decreased (*p*<0.05; *n*=4) the glucose, cholesterol, and triglyceride levels and also the liver and plasma malondaldehyde levels. Moreover, the SCFP slightly increased the reduced glutathione levels in the liver.

**Conclusion:**

The SCFP could be used to control the blood pressure of hypertensive subjects for a long period of time and could improve metabolic complications associated to cardiovascular diseases.

Some food compounds may exert biological activities, and nowadays there is a growing interest on functional foods that could control arterial blood pressure. Functional foods are foods that show healthy properties besides their nutritional value. In particular, the close relationship between the consumption of foods rich in antioxidants and the drop of arterial blood pressure has assumed great importance in recent times, emerging as a non-pharmacological antihypertensive strategy (1, 2).

Dietary fiber includes a group of food components, present in cereals, fruits, and vegetables, which are resistant to digestive enzymes. Attending to its water solubility, fiber can be classified into insoluble and soluble fiber (3). Dietary fiber has demonstrated beneficial effects on different cardiovascular risk factors such as obesity (4, 5), hyperglycaemia and diabetes mellitus type 2 (6, 7), and hypercholesterolemia (8, 9). Even though essential hypertension is a well-known risk factor for cardiovascular disorders, the effect of dietary fiber on arterial blood pressure has been studied less than its effect on the above-mentioned cardiovascular risk factors.

Recent research has been focused on cocoa and its products because of their antihypertensive properties that can be attributed to their high antioxidant polyphenol content (10, 11). Data show that sustained chronic consumption of cocoa products can lead to a relatively low accumulation of cocoa polyphenols in human plasma, sufficient to exert some health-relevant bioactivity (12). The aim of the study is to evaluate the antihypertensive properties of a soluble cocoa fiber product (SCFP) that was obtained by a patented enzymatic process from cocoa husk, in spontaneously hypertensive rats (SHR) (13). The SCFP had previously demonstrated beneficial effects on the body weight and the lipid profile of hypercholesterolemic Sprague-Dawley rats (14) and produced positive effects on hyperglycaemia, hypercholesterolemia and arterial blood pressure in Zücker fatty rats (15). Therefore, in this study, we have also investigated the effect of the long-term intake of SCFP on the body weight, glycaemia, cholesterol, and triglyceride plasma levels of the treated SHR. Furthermore, the redox status and the plasma angiotensin-converting enzyme (ACE) activity were also determined in these animals.

## Materials and methods

### Soluble cocoa fiber product

The chemical composition, polyphenol content, and antioxidant capacity of SCFP were characterized by Ramos et al. (14). Table 1 shows the more relevant information reported by these researchers for the present study.

**Table 1:** Composition of total, soluble and insoluble, fiber; total polyphenols, procianidine; and antioxidant capacity of soluble cocoa fiber product

Total dietary fiber (g/mg product)	46.50
Soluble fiber	42.80
Insoluble fiber	3.70
Total polyphenols (g/mg product)	2.24
Total procianidine	0.032
ORAC Total (µmol TE/g)	569

### Protocol in rats

Forty-eight 10-week-old male SHR, weighing 255–265 g, were purchased from Charles River Laboratories (Barcelona-Spain) for this study. These animals were caged in groups of four rats, at a temperature of 23°C, with 12 h light/dark cycles. They were in turn randomly divided into six groups of eight animals with *ad libitum* intake. Throughout the experimental period, the SHR of the established groups were fed a standard diet (A04 Panlab, Barcelona-Spain). From the 10th week of life until the animals were 20 weeks old (treatment period), the consumption of drinking fluids in these groups was as follows: tap water (neutral vehicle as negative control group), 200 mg/kg/day SCFP, 400 mg/kg/day SCFP, 800 mg/kg/day SCFP, 800 mg/kg/day beta-glucan 0.75 (BETA-G) (fiber reference group), and 50 mg/kg/day captopril (positive control group). From 20 to 24 weeks of life (follow-up period), all groups consumed tap water as a drinking fluid. Weekly body weight of all animals was recorded up to the 24th week of life. Daily intake of drinking fluids and freely accessible feed was also estimated weekly in the animals from the different groups throughout the experimental period. Four 20-week-old rats of each group were killed by decapitation. Blood and liver samples were quickly collected from these animals for future analytical evaluations. Glucose, total cholesterol, triglyceride, and malondialdehyde (MDA) levels were determined in the plasma samples. These samples were also used to establish the plasma antioxidant capacity and the plasma ACE activity of the SHR. Subsequently, MDA and reduced glutathione (GSH) concentrations were also measured in liver samples.

At the end of the experimental period, the 24-week-old rats were killed by decapitation, and the same tests and procedures recorded for the killed 20-week-old rats were applied to these animals as well.

The experiments were designed and performed in accordance with the European and Spanish legislation on care and use of experimental animals (2010/63/UE; Real Decreto 53/2013) and were approved by the Ethics Committee at the Universidad Complutense de Madrid.

### Blood pressure measurements

The systolic blood pressure (SBP) was measured weekly in the SHR by the tail cuff method with some modification as described in Miguel et al. (16), during all the experimental period. Before the measurements, the rats were kept at 38°C for 10–15 min to make the pulsation of the tail artery detectable. Arterial blood pressure measurements were performed at the same time of the day (between 9 a.m. and 1 p.m.) in order to avoid the influence of the circadian cycle, and the values of SBP were obtained by estimating the average reading of five measurements.

### Plasma and liver samples

The blood samples were collected in tubes containing lithium heparin as anticoagulant and centrifuged at 3,500*g* for 20 min to obtain the plasma. A piece of liver tissue was homogenized at 4°C in a potter with phosphate-buffered saline (PBS) (0.01 M PBS, 0.15 M NaCl, pH 7.4). The homogenates of liver were centrifuged at 5,000*g* for 15 min at 4°C, and the supernatant was recovered. Plasma samples and supernatants of liver samples were stored at −80°C until the analysis. Protein content in the liver samples was determined by the spectrophotometric technique (DC protein assay, Biorad, Michigan, USA). We used bovine serum albumin as standard (Sigma-Aldrich, Milwaukee, WI, USA).

### Determination of glucose, total cholesterol, and triglycerides

Glucose, total cholesterol, and the triglyceride levels in the plasma of the animals were determined using colorimetric assays from Spinreact (Girona, Spain), according to the manufacturer instructions. The plasma values were expressed as mg/dL.

### Determination of plasma antioxidant capacity

The antioxidant capacity of the plasma samples was determined using the oxygen radical absorbance capacity-fluorescein (ORAC) assay, previously described by Manso et al. (17). The final assay mixture (200 µl) contained 20 µl of the plasma samples or 20 µl of Trolox (Sigma-Aldrich, St. Louis, MO, USA) [6-hydroxy-2,5,7,8-tetramethylchroman-2-carboxylic acid] at different concentrations (1–8 µM) as standard. Samples and standards were dissolved in 75 mM PBS (pH 7.4). Disodium fluorescein (70 nM) (Sigma-Aldirch Log, Schnelldorf, Germany) as oxidizable substrate was prepared using the same buffer and stored in dark conditions at 4°C for a maximum of 4 weeks, and 2,2′-azobis (2-methylpropionamidine) (AAPH) (12 mM) (Sigma-Aldrich, Milwaukee, WI, USA) was used as oxygen radical generator. The AAPH solution was prepared using 75 mM PBS (pH 7.4) just before the analysis. For the blank, the plasma was substituted by 20 µl of PBS. A Polarstar Galaxy plate reader (BMG Labtechnologies GmbH, Offenburg, Germany) with a 485-P excitation and a 520-P emission filter was used. The fluorescent plate reader was controlled by the Fluostar Galaxy software version (4.11-0). Black 96-well microplates (96F untreated microwell, NuncTM, Denmark) were used. Fluorescence measurement was carried out at 37°C and was recorded every minute for 40 min. All reaction mixtures were prepared in duplicate, and at least three independent runs were performed for each sample. ORAC values were calculated and expressed as µmol Trolox/ml plasma.

### Determination of malondialdehyde

Plasma and liver MDA values were measured by a thiobarbituric acid (TBA) assay, previously described by Rodríguez-Martínez and Ruiz-Torres (18) and modified by Quiñones et al. (19). Plasma and hepatic tissue samples were incubated with NaOH for 30 min, and the samples were then mixed with 20% trichloroacetic acid in 0.6 M HCl (1:1, v/v). The tubes were kept on ice for 20 min to precipitate components and to avoid possible interferences. The samples were centrifuged at 1,500*g* for 15 min before adding TBA (120 mM in Tris 260 mM, pH 7) to the supernatant in a proportion of 1:5 (v/v); then, the mixture was boiled at 97°C for 30 min. Spectrophotometric measurements at 535 nm were made at 20°C. The plasma MDA values were expressed as nmol MDA/ml plasma, and the liver MDA values were expressed as nmol MDA/mg tissue protein.

### Determination of reduced glutathione

The reduced glutathione (GSH) levels in the liver were determined using the monochlorobimane (mCB) method described by Kamencic et al. (20). The final assay mixture (100 µl) contained 90 µl of the liver samples or of reduced glutathione as standard (Sigma-Aldrich Chemie CMBh, Germany) in a PBS solution (pH 7.4) at different concentrations (0.001–10 Mm) and 10 µl of glutathione *S*-transfer (1 U/ml) obtained from equine liver and monochlorobimane (1 mM) (Fluka Biochemical, Swaziland). The samples were then allowed to incubate in dark at room temperature for 30 min. The GSH–mCB adduct was measured using a Labsystems Fluoroskan II microtiter reader with excitation at 380 nm and the emission was measured at 470 nm. The liver GSH levels were expressed as µmol GSH/µg tissue protein.

### Determination of ACE activity

The ACE activity in the plasma samples was measured by a fluorimetric method as explained by Miguel et al. (21).Then, triplicate plasma (3 µl) was incubated for 15 min at 37°C with 40 µl of assay buffer containing the ACE substrate 5 mM Hip-His-Leu (Sigma). The reaction was stopped by the addition of 190 µl of 0.35 N HCl. The product generated, His-Leu, was measured fluorimetrically following 10 min incubation with 100 µl of 2% *o-*phthaldialdehyde in methanol. Fluorescence measurements were carried out at 37°C in a Fluostar Optima plate reader (BMG Labtech, GmbH, Offenburg, Germany) with 350-nm excitation and 520-nm emission filters. The fluorescent plate reader was controlled by the Fluostar Optima software. Black 96-well polystyrene microplates (Biogen Científica, Madrid, Spain) were used. A calibration curve with ACE from rabbit lung (Sigma, St. Louis, MO) was included in each plate. The ACE activity was expressed as mU ACE/ml plasma.

### Statistical analysis

The results are expressed as mean values±standard error of the mean (SEM) for a minimum of four rats and were analyzed by a one- or two-way analysis of variance (ANOVA) and the differences between the groups were assessed by the Bonferroni test and Student t test, using the GraphPad Prism software. We considered as the treatment period the time elapsed from the 10th until the 20th week of life and we considered the follow-up period from the 20th to the 24th week of life. Differences between the means were considered to be significant when the p values were <0.05.

## Results

### Arterial blood pressure

**Fig. 1:**
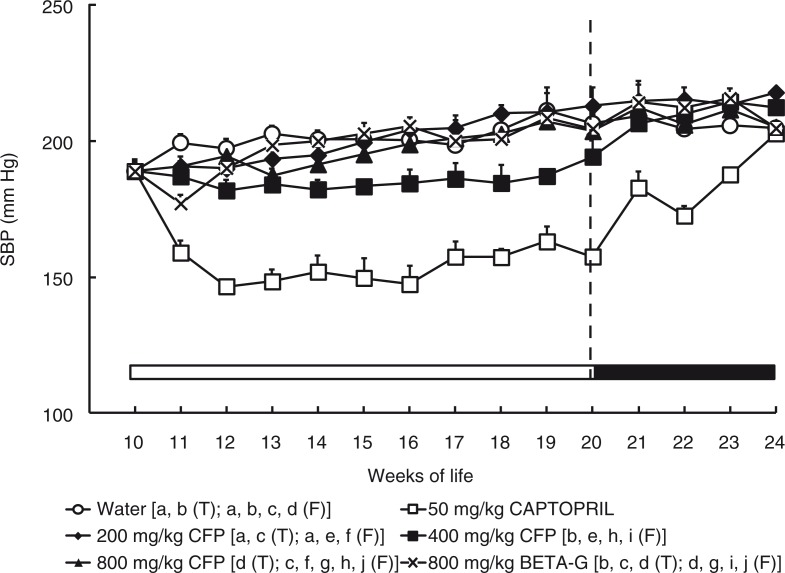
Systolic blood pressure (SBP) of spontaneously hypertensive rats. The animals consumed different fluids from the 10th week of life until the 20th weeks of life (T=treatment period, indicated by a white bar) and received different daily treatments: tap water (○), 50 mg/kg/day captopril (□), 200 mg/kg/day soluble cocoa fiber product (SCFP) (♦), 400 mg/kg/day SCFP (■), 800 mg/kg/day SCFP (▲), and 800 mg/kg/day beta-glucan 0.75 mm (BETA-G) (x). The rats consumed tap water from the 20th week of life until the 24th week of life (F=follow-up period, indicated by a black bar). Data are mean values±SEM for 8 animals in T and for 4 animals in F. *p* estimated by two-way ANOVA and Bonferroni test (*p*<0.05). Similar letters represent no statistical differences.

The SBP of the different groups of SHR are shown in Fig. 1. SHR of the negative control group that consumed tap water experienced a gradual increase in SBP from the start of the study, when the animals were 10 weeks old, which reached maximal values at about 19–20 weeks of life. From this age, the SBP of these rats remained constantly high and presented similar values until the 24th week of life. Hypertension was decreased in the SHR groups treated with 50 mg/kg/day captopril or with 400 mg/kg/day SCFP. From the 10th to the 20th week of life (treatment period), the lowest values of SBP were observed in the rats of the positive control group that were treated with captopril. The dose of 400 mg/kg/day of SCFP also decreased hypertension in SHR. This group showed lower values of SBP than the animals from the negative control group. However, the animals treated with 200 mg/kg/day SCFP 800 mg/kg/day SCFP or 800 mg/kg/day BETA-G did not show any antihypertensive effect, and the values of SBP in these groups were quite similar to those of the negative control group. The SHR that had been treated with 400 mg/kg/day SCFP showed an increase in SBP when they were given, at the 20th week of life, tap water as drinking fluid. Moreover, these animals achieved SBP values similar to those of the negative control group during the follow-up period. SBP also increased in the SHR that had received captopril when the pharmacological treatment finished, but in this case the reversion of the antihypertensive effect was less abrupt than when the treatment with 400 mg/kg/day SCFP was disrupted (Fig. 1).

Body weight increased progressively in all groups of rats from the beginning of the study, and throughout the experimental period, the growth was quite similar in the SHR treated with SCFP or BETA-G and in the negative control rats. However, from the 15th weeks of life, the body weight in the captopril group was slightly lower than in the remaining groups (data not shown). Food intake was also very similar in all groups along the experimental period (data not shown), but some differences could be observed in the fluid intake, even if it usually varied in all animals. In this context, we can stand out that the fluid intake in the SHR treated with 50 mg/kg/day captopril or 800 mg/day BETA-G was higher than that in the other rats (*p*<0.05). When these treatments were disrupted, a reduction in the fluid intake was observed in the rats; however the animals that had been treated with 800 mg/day BETA-G maintained a drinking behavior slightly more pronounced than those of the other groups until the end of the experimental period (data not shown).

### Glycaemia and lipid profile

**Fig. 2:**
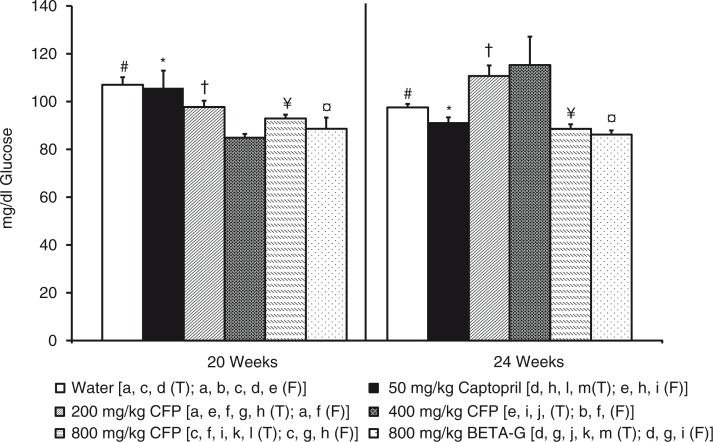
Histograms of plasma glucose levels (mg/dl) from spontaneously hypertensive rats. The animals consumed different fluids from the 10th week of life until the 20th weeks of life (T=treatment period) and received different daily treatments: tap water (□), 50 mg/kg/day captopril (■), 200 mg/kg/day soluble cocoa fiber product (SCFP) (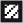), 400 mg/kg/day SCFP (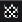), 800 mg/kg/day SCFP (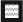), and 800 mg/kg/day beta-glucan 0.75 mm (BETA-G) (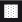). The rats consumed tap water from the 20th week of life until the 24th week of life (follow-up period=F). Data are mean values±SEM for 8 animals in T and for 4 animals in F. *p* estimated by one-way ANOVA and Bonferroni test between data from different groups, and by student t test on the same group at the end of T and at the end of F (*p*<0.05). Similar letters or symbols represent no statistical differences.

Plasma glucose was lower in the SHR that had been treated with 400 mg/kg/day SCFP or 800 mg/kg/day BETA-G than in the SHR that consumed tap water (negative control group). The animals treated with the remaining doses of SCFP also showed a slight decrease in glycaemia when compared with the negative control group, but no significant differences were appreciated in these comparisons. In addition, plasma glucose in the SHR treated with 50 mg/kg/day captopril was similar to plasma glucose in the negative control group. After the follow-up period, the glycaemia differences mentioned above could not be observed. Nevertheless, plasma glucose significantly increased in the animals that had been treated with 400 mg/kg/day SCFP when the treatment was disrupted (Fig. 2).

**Fig. 3:**
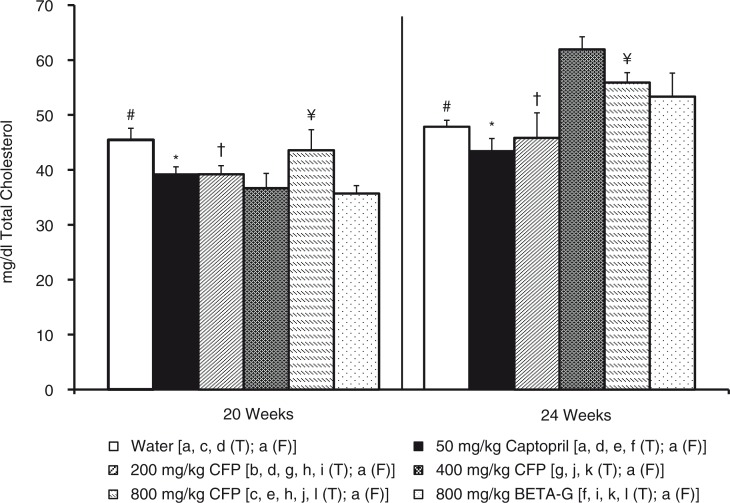
Histograms of plasma total cholesterol levels (mg/dl) from spontaneously hypertensive rats. The animals consumed different fluids from the 10th week of life until the 20th weeks of life (T=treatment period) and received different daily treatments: tap water (□), 50 mg/kg/day captopril (■), 200 mg/kg/day soluble cocoa fiber product (SCFP) (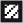), 400 mg/kg/day SCFP (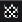), 800 mg/kg/day SCFP (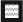), and 800 mg/kg/day beta-glucan 0.75 mm (BETA-G) (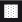). The rats consumed tap water from the 20th week of life until the 24th week of life (F=follow-up period). Data are mean values±SEM for 8 animals in T and for 4 animals in F. *p* estimated by one-way ANOVA and Bonferroni test between data from different groups, and by student t test on the same group at the end of T and at the end of F (*p*<0.05). Similar letters or symbols represent no statistical differences.

**Fig. 4:**
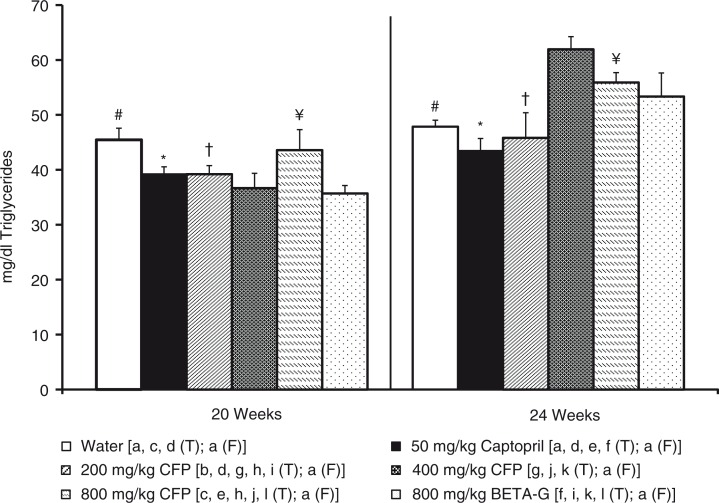
Histograms of plasma triglyceride levels (mg/dl) from spontaneously hypertensive rats. The animals consumed different fluids from the 10th week of life until the 20th weeks of life (T=treatment period) and received different daily treatments: tap water (□), 50 mg/kg/day captopril (■), 200 mg/kg/day soluble cocoa fiber product (SCFP) (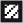), 400 mg/kg/day SCFP (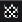), 800 mg/kg/day SCFP (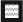), and 800 mg/kg/day beta-glucan 0.75 mm (BETA-G) (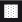). The rats consumed tap water from the 20th week of life until the 24th week of life (F=follow-up period). Data are mean values±SEM for 8 animals in T and for 4 animals in F. *p* estimated by one-way ANOVA and Bonferroni test between data from different groups, and by student t test on the same group at the end of T and at the end of F (*p*<0.05). Similar letters or symbols represent no statistical differences.

Total plasma cholesterol and plasma triglycerides were also lower in the SHR that had been treated with 400 mg/kg/day SCFP or 800 mg/kg/day BETA-G, than in the group that consumed tap water (negative control group). No differences were observed in these parameters in the SHR that had received the remaining treatments and the rats that were not treated. In accordance with glucose results, total cholesterol and triglycerides in the plasma significantly increased in the animals that had been treated with 400 mg/kg/day SCFP or 800 mg/kg/day BETA-G when the treatment finished (Figs. 3 and 4).

### Redox status

**Fig. 5:**
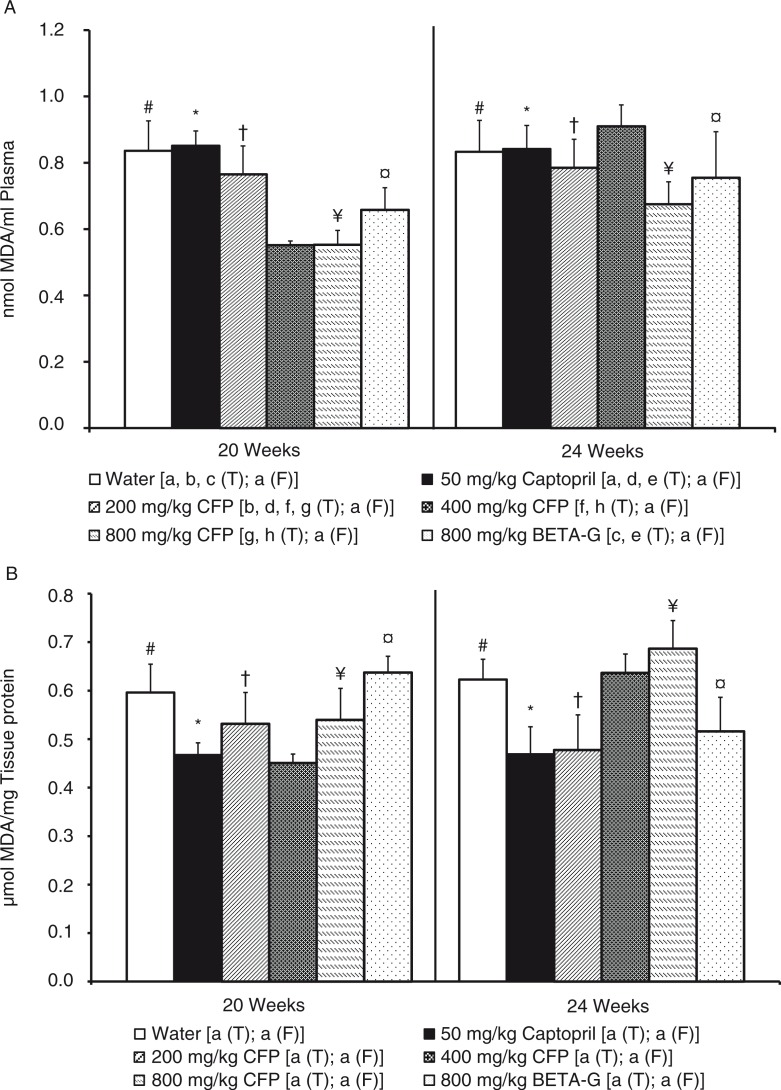
Histograms of (A) plasma malondialdehyde (nmol MDA/ml plasma), and (B) liver malondialdehyde (µmol MDA/mg tissue protein) from spontaneously hypertensive rats. The animals consumed different fluids from the 10th week of life until the 20th weeks of life (T=treatment period) and received different daily treatments: tap water (□), 50 mg/kg/day captopril (■), 200 mg/kg/day soluble cocoa fiber product (SCFP) (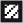), 400 mg/kg/day SCFP (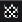), 800 mg/kg/day SCFP (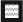), and 800 mg/kg/day of beta-glucan 0.75 mm (BETA-G) (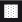). The rats consumed tap water from the 20th week of life until the 24th week of life (F=follow-up period). Data are mean values±SEM for 8 animals in T and for 4 animals in F. *p* Values estimated by one-way ANOVA and Bonferroni test between data from different groups, and by student t test on the same group at to the end of T and at the end of F (*p*<0.05). Similar letters or symbols represent no statistical differences.

No differences were observed in plasma ORAC values and in liver reduced glutathione levels in the rats, neither at the end of the treatment period (20th week of life) nor at the end of the follow-up period (24th week of life) (data not shown). However, plasma MDA levels were lower in the SHR that had been treated with 400 mg/kg/day SCFP or 800 mg/kg/day SCFP, than in the SHR that consumed tap water (negative control group). Nevertheless, the plasma MDA levels in the SHR treated with 50 mg/kg/day captopril, 200 mg/kg/day SCFP or 800 mg/kg/day BETA-G, showed no significant differences when compared with the negative control group. When the corresponding treatments were disrupted, the plasma MDA levels of the groups that had been treated with 400 mg/kg/day SCFP or 800 mg/kg/day SCFP increased, and, at the 24th week of life, this biomarker was similar in all the animals (Fig. 5a). Liver MDA levels were somewhat lower in the SHR treated with 50 mg/kg/day captopril or 400 mg/kg/day SCFP, than in the control SHR, even if the differences did not attained significant relevance. When captopril treatment was disrupted, the rats maintained decreased levels of liver MDA, but, after the follow-up period, liver MDA levels increased in the rats that had been treated with 400 mg/kg/day SCFP. In fact, at the 24th week of life, this biomarker was similar in these animals and in the SHR of the control group (Fig. 5b).

### Plasma ACE activity

The ACE activity was significantly higher in the plasma of the SHR that had been treated with 50 mg/kg/day captopril than in the control group, but the plasma activity of this enzyme decreased in the captopril group when this treatment was disrupted. In fact, the plasma ACE activity of the rats that had been treated with captopril was similar to the corresponding values in the control rats at the end of the follow-up period. A slight, but not significant, increase of the plasma ACE activity was also observed in the rats treated with 800 mg/kg/day SCFP or 800 mg/kg/day BETA-G, and the animals of these groups showed also a decrease in the plasma activity of this enzyme when these treatments finished (data not shown).

## Discussion

Fiber components organize functions of the large intestine and have important physiological effects on glucose, lipid metabolism, and mineral bioavailability (22). However, as mentioned earlier, the effect of dietary fiber on arterial blood pressure has not been clearly established. Hypertension is a chronic pathology that requires chronic treatment, and the long-term administration of functional products without side effects is an attractive possibility for the patients with this pathology. In this context, we have studied the long-term effect of the SCFP in SHR.

The development of hypertension in SHR is very similar to that in humans (23) and, nowadays, these animals are considered the best experimental model to evaluate antihypertensive functional food ingredients (24). There is an initial period in the life of the SHR in which arterial blood pressure clearly increases (23, 25). Nevertheless, from the 10th week of life, the SHR show more stable values of this variable. The present study was carried out with 10- to 24-week-old SHR. Along this period, we could observe that captopril, and also SCFP, clearly decreased SBP in these animals. As expected, the drug was more effective in reducing arterial blood pressure than the fiber product, but it is important to notice that captopril is an antihypertensive agent with recognized clinical efficacy. Moreover, our research group characterized the dose of captopril in this study (50 mg/kg/day) as a high dose that causes the maximum effect when it was short term administered to the SHR (data not shown). In addition, we want to highlight that the decrease in SBP caused by 400 mg/kg/day SCFP was consistent throughout the treatment period. In contrast, the decrease in SBP could not be observed in the rats treated with BETA-G.

As Table 1 shows, the SCFP contains polyphenols and we believe that these compounds could be the agents responsible for the antihypertensive effect of this fiber product. It should be noted that the increase in the dose of SCFP was not always accompanied with an increased antihypertensive effect in the SHR. In particular, the highest dose of SCFP (800 mg/kg/day) caused minimal effect in these animals than the 400 mg/kg/day dose. This paradox of a no dose-dependent antihypertensive effect had been observed earlier by our research group with different polyphenols and polyphenol-rich products, which we have evaluated also in SHR (26, 27). Polyphenolic compounds act as natural antioxidants and have interesting benefits to health, but high doses of polyphenols might not always be needed to manifest beneficial biological activities (28). In addition, several studies show controversial results of exogenous antioxidants, debating the type, dosage, and matrix of these compounds that determine the balance between beneficial and deleterious effects (29), and it is known that polyphenols can even act as pro-oxidants under certain conditions, such as high doses or the presence of metal ions (29, 30).

We should discard in any case that other possible compounds of SCFP that differ from polyphenols could be implicated in the antihypertensive effect of this fiber product. It is known that cocoa products are also rich in minerals, such as magnesium, potassium, and calcium, which have antihypertensive properties (10, 11, 31), but the SCFP did not contain significant quantities of these minerals. SCFP contains theobromine, and theobromine relaxes arterial tissue, but we could also discard this xanthine as the main responsible agent for the antihypertensive effect of SCFP. As we hare mentioned, the effect of SCFP is not dose-dependent but the arterial effects of theobromine are dose-dependent (32). Likewise, it has been reported that theobromine form cocoa does not affect blood pressure in healthy and hypertension type I subjects (33).

When captopril and SCFP treatments were disrupted, arterial blood pressure rose gradually in the rats. This confirms the antihypertensive properties of these treatments. In fact, at the end of the follow-up period, the SBP values were similar in all groups of the SHR. Having in mind the marked effect of captopril, it was not surprising to observe that the reversion of the antihypertensive effect was slower in the group that had received this drug than in the group that had been treated with the SCFP.

The SHR treated with captopril gained weight at a lower rate than did other animals, and these findings agree with previous studies showing that blockade of ACE system slows body weight gain in this rat strain (34).

We have observed that food intake was similar in all animal groups. Nevertheless, liquid intake increased markedly in the rats that had ingested the captopril solution. This is not surprising because it has been demonstrated that ACE inhibitors induce thirst and increase water intake and urine output in rats (35). The animals treated with the highest dose of fiber (800 mg/kg/day CFP or 800 mg/kg/day BETA-G) also drank more than the other animal groups. These data could be related with the water requirements of fiber to form gels.

According to the European Food Safety Agency, beta-glucan from any cereal maintains lower cholesterol levels and decreases plasma glucose levels (36). For these reasons, we have used BETA-G as a standard fiber to study these healthy effects. We demonstrated that BETA-G and SCFP reduced glycaemia in the treated SHR with 20 weeks of life. In addition, we showed that both products reduced plasma cholesterol and triglyceride levels in these animals. Researchers have demonstrated that dietary fiber has beneficial effects on hyperglycaemia and the lipid profile (37–39), but the fiber product that we have studied could be, in particular, useful in the subjects in which these metabolic complications were associated with elevated arterial blood pressure.

It is well known that antioxidant therapy has positive results on hypertensive patients (10, 40). Therefore, in this study, we evaluated the plasma antioxidant capacity of the rats after the different treatments. We have also analyzed MDA in the plasma and the liver of the rats because this metabolite is a biomarker that enabled us to estimate lipid peroxidation. In addition, GSH, an important antioxidant tripeptide that prevents damage caused by free radicals and peroxides, was also measured in the rats. The most effective antihypertensive dose of SCFP (400 mg/kg/day) significantly decreased plasma and liver MDA values. As in the case of arterial blood pressure, MDA also returned to basal values when this treatment finished. Therefore, the SCFP could attenuate plasma-transported lipids and their peroxidation, improving therefore the cardiovascular conditions. Our research group demonstrated that the short-term administration of SCFP decreases both arterial blood pressure and the ORAC values of the SHR (unpublished data). Nevertheless, we did not obtain significant results in the ORAC and GSH determinations of our rats perhaps because the prolonged period of treatment sets up pro-oxidant compensatory mechanisms.

ACE is critical for blood pressure control, and specific inhibitors of this enzyme are used as antihypertensive drugs. Polyphenols can inhibit this enzyme *in vitro* (41, 42). To better elucidate the antihypertensive mechanism of the SCFP, we have also verified the plasma ACE activity in all rat groups. The plasma ACE activity was significantly higher in the 50 mg/kg/day captopril treated animals than in the remaining SHR. It is important to highlight that long-term administration of ACE inhibitors causes an increase in plasma ACE (43–45) and also that the ACE activity in the vascular tissue of SHR increases in particular during the long-term treatment with inhibitors of this enzyme (46). A slight, but not significant, increase of the plasma ACE activity was also observed in the rats treated with the highest dose of SCFP, but more studies are needed to clarify if SCFP polyphenols may act by inhibiting ACE.

In conclusion, long-term intake of SCFP improves SBP, glycaemia, and lipid profile in SHR. The SCFP also decrease lipid peroxidation in these animals. All these effects could indirectly promote a decrease in arterial blood pressure, but we should not forget that this fiber product contains polyphenols, and that these agents have demonstrated antioxidant properties and antihypertensive effect. Therefore, fiber products could benefit hypertension and related metabolic diseases, and the SCFP could in particular benefit this pathology. This study supports polyphenols are that responsible, at least in part, for the SCFP antihypertensive effect, and suggest, after appropriate clinical trials, to consider this product as a possible functional food ingredient for hypertensive patients.
